# Duration of immunity to SARS-CoV-2 in children after natural infection or vaccination in the omicron and pre-omicron era: A systematic review of clinical and immunological studies

**DOI:** 10.3389/fimmu.2022.1024924

**Published:** 2023-01-11

**Authors:** Danilo Buonsenso, Francesca Cusenza, Lucrezia Passadore, Francesca Bonanno, Claudia De Guido, Susanna Esposito

**Affiliations:** ^1^ Department of Woman and Child Health and Public Health, Fondazione Policlinico Universitario A. Gemelli Istituto di Ricovero e Cura a Carattere Scientifico (IRCCS), Rome, Italy; ^2^ Centro di Salute Globale, Università Cattolica del Sacro Cuore, Roma, Italy; ^3^ Pediatric Clinic, Department of Medicine and Surgery, University of Parma, Parma, Italy

**Keywords:** COVID-19, SARS-CoV-2, vaccine, immunity, children

## Abstract

**Background:**

Duration of humoral and cellular memory in children previously infected SARS-CoV-2 or vaccinated and subsequent risk of reinfection is still not fully elucidated.

**Methods:**

Systematic review of studies retrieved from medical databases and article reference lists.

**Results:**

From 2420 identified articles, 24 met the inclusion criteria. Children infected during the pre-omicron era developed long lasting (at least 10-12 months) humoral and cellular immunity against pre-Omicron SARS-CoV-2 variants, but have reduced *in vitro* cross-reactivity against Omicron. Conversely, although vaccination has a limited efficacy in preventing new infection with pre-Omicron and Omicron variants, *in vitro* studies suggested that vaccine-induced immunity provides better *in vitro* cross-neutralization against pre-Omicron and Omicron variants. Preprints published after the period of inclusion of our review suggested that overall risk of infection after Omicron infection is reduced, but children developed weak neutralizing responses in about half cases.

**Conclusions:**

Available evidence, although limited, suggested a long-lasting but unperfect protection of previous infections or vaccination against pre-Omicron and Omicron variants. Based on our findings, it might be reasonable to offer families of children infected before Omicron a booster vaccination. A similar indication should be proposed also for those infected with Omicron, specifically for more fragile children at higher risk of COVID-19-related complications, based on better cross-variant neutralisation induced by vaccination.

**Systematic review registration:**

PROSPERO, identifier ID 353189.

## Graphical Abstract

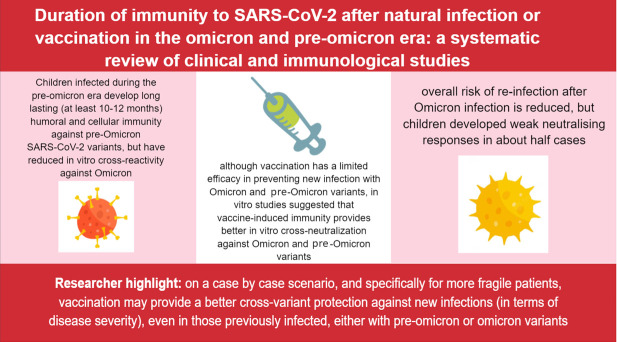

## Introduction

Since the first description of SARS-CoV-2, the pandemic has significantly evolved through different phases in terms of epidemiologic, clinical and virologic perspectives. Initially, the clinical impact of Covid-19 on adults has been massive and healthcare systems worldwide have struggled in providing support to the massive number of patients requiring admission. Initially, children had been relatively spared from the pandemic and represented a minimal percentage of cases (1), particularly of the most severe ones (2),. Later, several different variants have emerged with improved transmissibility and different ability to cause severe disease (3), and the number of children diagnosed with Covid-19 or requiring hospitalisation increased significantly, although the number of critical diseases remained low. Importantly, while the number of MIS-C cases also dropped, it has become evident that also children could develop Long COVID after SARS-CoV-2 infection (4).

In the meantime, different vaccines have been produced, which are effective against severe disease, although efficacy in preventing transmission is low, particularly since the Omicron era. Although the same efficacy has been demonstrated in children in the short time follow-up of initial trials run by drug companies (5), a rare but real complication in young males - acute myocarditis - have been documented by several independent reports (6). This side effect, along with the observation that most children developed a mild or asymptomatic disease, led some authors or countries to question the need of vaccinating children, as a less clear benefit-risk ratio can be demonstrated in children compared with adults (7). To make the scenario even more complicated, a huge number of children have been infected during Omicron wave and authors have claimed that previous infection would confer enough immunity to protect against severe disease in case of reinfections, therefore further questioning the utility of COVID-19 vaccines in children.

Given these rapidly evolving scenarios and growing and fast amount of publications, in order to better understand the risks of COVID-19 after previous infections or vaccination, we performed a systematic review of available studies that assessed the duration of immunity after disease or vaccination in children, evaluating clinical information (clinically evident reinfection) and immunological findings (duration of humoral or cellular immunity). Such an approach can provide better evidence and information on how to organise vaccination campaigns in the current context of massive SARS-CoV-2 circulation, but also a general better understanding on the development of immunity after respiratory infections.

## Methods

Our systematic review and meta-analysis was performed along with the Preferred Reporting Items for Systematic Reviews (PRISMA) extension for scoping reviews (**Supplementary Table S1**) (8). The protocol of this systematic review and meta-analysis was registered in the International Prospective Register of Systematic Reviews (PROSPERO) database (ID 353189).

### Search strategy

The literature search strategy (**Supplementary Table S2**) was aimed at identifying those clinical studies evaluating duration of cellular mediated or humoral immunity developed after SARS-CoV-2 natural infection or vaccination in the paediatric population.

The systematic search was conducted according to the following PICOS approach: Population: paediatric patients who have been vaccinated against SARS-CoV-2 or got infected by SARS-CoV-2; Intervention: evaluation of cellular mediated or humoral immunity; Comparison between immunity after natural infection and immunity after vaccination; Outcomes: duration of immunity after natural infection and after vaccination against SARS-CoV-2; Study design: observational cohort studies (either prospective or retrospective).

A systematic search of PubMed was performed from March 7th until April 6th, 2022. Additional relevant studies were also identified by hand-searching reviews on the topic and exploring the list of references of selected papers.

Since a number of relevant preprints have been published after the conclusion of our systematic review specifically addressing the immunological responses and risk of infection during and after the Omicron waves, we performed a search update on July 13th, 2022 and relevant papers have been subsequently included in an updated results section.

### Eligibility criteria and identification of studies

The systematic review included only clinical studies aimed at determining the duration of cellular mediated or humoral immunity after natural infection or vaccination against SARS-CoV-2, or the cases of reinfections after previous infection or vaccination.

The analysis included observational cohort studies either prospective or retrospective, all including children aged <18 years. Studies that do not assess the duration of immunity after infection/vaccination and those without available paediatric data were excluded, as well as reviews, meta-analysis, editorials, and case series/reports. Papers written in a non-European language were excluded. All *in vitro* and animal experimental studies were also excluded.

### Study selection

All studies published between February 1st, 2021, and July 13th, 2022 were considered (N= 2420). To enhance consistency, all reviewers (four residents and one senior) screened the same numbers of publications, discussed results, and amended the screening and data extraction before beginning screening for this review. The same five reviewers, working in pairs, assessed the titles, abstracts and full text of all publications identified by the search. Disagreements on study selection and data extraction were solved by consensus and discussion with other reviewers, if needed.

### Outcome measures

The major outcome of interest was evaluating duration of immunity, developed by paediatric patients after SARS-CoV-2 natural infection or vaccination, with a distinction between:

(i) duration of cellular mediated immunity developed after SARS-CoV-2 natural infection or vaccination;
(ii) duration of humoral immunity developed after SARS-CoV-2 natural infection or vaccination;

The secondary outcome comprised:

(i) number of infections and disease severity after vaccination or after natural infection;
(ii) neutralizing activity of cellular mediated or humoral immunity against variants;
(iii) specific comments on T and B compartment of cellular immunity.

### Data extraction

Four reviewers independently analyzed the studies included in the review, extracting data related to patients and methods characteristics, duration of cellular mediated or humoral immunity, clinical results and reported outcomes. Results were checked again, across the original manuscript, by a fifth researcher.

### Data synthesis

Characteristics of the included (and excluded) studies were presented in tabulated form on an Excel sheet. The data were collected in columns: study citation; year; study country; number of children included; age of group; the sex of the group; study population (healthy, comorbidities, both); possible comorbidities of the study’s pediatric population; duration of immunity after vaccination or infection; methods of study; if vaccination, specify which vaccine; if infection, specify which variants (if known) or wave (report study period); length of follow-up; in clinical findings: number of infections and disease severity after vaccination or after natural infection; in humoral immunity: main findings, specific comments on neutralizing antibodies and on neutralizing activity against variants; in cellular immunity: main findings, specific comments on B cell or T cell compartment and on activities against variants; study limitation and other comments.

### Quality assessment in individual studies

The quality of included studies with comments about study limitations, including whether children’s ages would be translated into specific age groups for analysis, were assessed by two reviewers (**Supplementary Table S3**).

None of the quality assessors were blinded.

## Results

### Study selection and description (synthesis of results)

**Figure 1:**
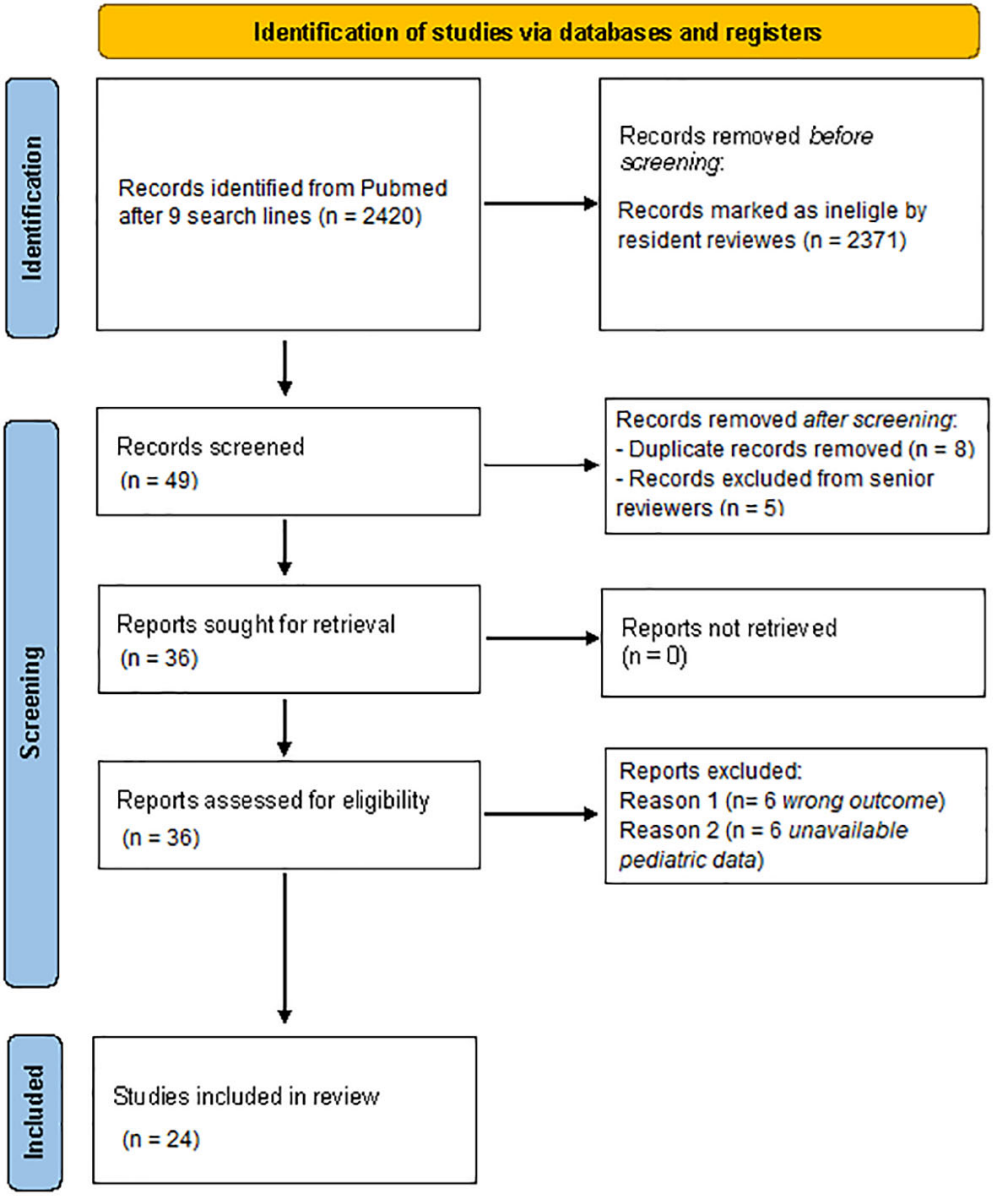
Flowchart of the study selection according to the PRISMA guidelines. Performed following “Page MJ, McKenzie JE, Bossuyt PM, Boutron I, Hoffmann TC, Mulrow CD, et al. The PRISMA 2020 statement: an updated guideline for reporting systematic reviews. BMJ 2021;372:n71. Doi: 10.1136/bmj.n71”.

Of the 2420 identified articles, 2371 were initially excluded following review of the titles and/or abstracts. Eight duplicates were removed after comparison of the different searches. At a second review, five more articles were excluded based on the abstract by a senior reviewer, and further 6 studies were excluded due to wrong interventions or outcomes, and six more studies for not including paediatric patients. Ultimately, 24 articles were included in the review (**Figure 1**).

The main demographic and clinical characteristics of the enrolled cohorts, aims and outcomes for each study are presented in **Table 1**; the main findings of each study assessing the duration of humoral and cellular immune responses after infections are presented in **Tables 2** and **3** respectively. Cases of microbiologically confirmed reinfections were only analyzed in vaccine efficacy studies, and are summarized in **Table 4**, along with immunological analyses.

**Table 1:** Main characteristics of the included studies.

Citation	Age groups Median age (years) (Ranges) (years)	Female Gender	Number of children	Study Population	Aim: Duration of immunity after vaccination or infection	Studied Reinfections and/or immunology	Study period	Length of Follow-up after infection/vaccination
Bloise et al, 2021 (9)	13.37 (9.6–14.3)	41.6%	12	H/C	infection	immunology	Between May 2020 and October 2020	180 days
Toh et al, 2021 (10)	4 (0–18)	Not specified	22	H	infection	immunology	Between May 2020 and October 2020	188–213
Interiano et al, 2021 (11).	0-19	Not specified	41	H	infection	immunology	End of April 2020	0->120 days
Ireland et al, 2021 (12)	8 (6-10)	50.5%	835	H	infection	immunology	from 18 October 2020 to 12 January 2021	1 month up to 6 month
Mayanskiy et al, 2021 (13)	8.2	39%	18	C	infection	immunology	from April 2020 to February 2021	18 weeks
Breuer et al, 2021 (14)	4.4 3 months - 18 years of age	44%	1138	H/C	infection	immunology	Between May 1, 2020, and July 31, 2020	4 months
Oygar et al, 2021 (15)	9.5 (0-17)	52%	84	H	infection	immunology	Since October 2020	IgG response: 14 days–3 months and 7-9 months; IgA response: 4 days up to 3 months.
Garrido et al, 2022 (16)	11.5 (5.2–16.5)	51%	69	H/C	infection	immunology	February 2020–September 2020	4 months
Messiah et al., 2021 (17)	12 (5-19)	53.6%	218	H/C	infection	immunology	study period: between 1st December 2020 to 31st March 2021	8 months
Han et al, 2022 (18)	9 (0-18)	36.8%	114	H	infection	immunology	from June to mid July 2020	84 days
Tsang et al, 2022 (19)	12 (2.7-18)	54.8%	31	H	infection	immunology	August 2020	29-219 days
Dowell et al, 2022 (20)	(10-13)	33%	35	H	infection	immunology	June 2020–July2021	2-12 months
Kinoshita et al, 2021 (21)	(<12)	33%	3	H/C	infection	immunology	2020-2021	65–84 days
Cotugno et al, 2021 (22)	6.8	38%	66	H	infection	immunology	March to April 2020	7 days (± 2 days)
Kaaijk et al, 2022 (23)	12 (2-16)	43.24%	37	H	infection	immunology	March–May, 2020	10 months
Cohen et al, 2021 (24)	7.8 (1.92-13)	54%	24	H	infection	immunology	Not specified	138 days
Frenck et al., 2021 (25)	(12-15)	49%	2260	H/C	vaccination	Infections and immunology	October 15, 2020, and January 12, 2021	21 days between the 2 doses of vaccine + 7 days after dose 2
Qin CX, et al. (26)	14 (12-18)	60%	57	C	vaccination	Infections and immunology	April-August 2021	two weeks after vaccine 1 (post-V1), and one month after vaccine 2 (post-V2)
Price A et a, 2022 (27)	15 (12-17)	48%	1185	H/C	vaccination	Infections	July 1, 2021–February 17,2022 (delta and omicron variant periods)	44 weeks
Powell A et al, 2022 (28)	14.5 (12-15 and 16-17)	50.8%	842929	H	vaccination	Infections	September 2021–Decembre 2021	≥105wks after vaccine 1 (post-V1), and ≥70wks after vaccine 2 (post-V2)
Fowlkes A et al, 2022 (29)	10 (5–15)	52%	1364	H/C	vaccination	Infections	July 25,2021–February 12, 2022	≥5 months after second vaccine dose receipt
Burns et al, 2022 (30)	14 (12–19)	59%	77	H	vaccination	Infections	May 2021–January 2022	6 months
Chen et al, 2022 (31)	Vaccinated group 15 (12.7-17.9) Recovered group 9.6 (2.6-17.9)	32.6%	49	H	vaccination compared to infection	Immunology	November 2020–January 2021	124 days
Haskin et al, 2021 (32)	Vaccinated group (13.5–26.8) Control group (10–20.6)	44%	38	C	vaccination compared to infection	Infections and immunology	Between February 20, 2021, and June 6, 2021.	4 months after vaccination; 10 months after Covid-19

H, healthy; C, comorbidities; H/C, both healthy children or with comorbidities.

**Table 2:** Findings on humoral immunity after infection.

Citation	Main findings	Specific comments on neutralizing antibodies
Bloise et al, 2021 (9)	At T30, 100% of children had positive IgG levels; at T180, 75% had positive IgG levels.	Not studied.
Toh et al, 2021 (10)	By day 43 (range 27–79), 15/19 (79%) children seroconverted and remained seropositive for more than 90 days. By day 195 (≈6 months), 14/17 (82%) of children were seropositive; from day 43 to 195, geometric mean antibody concentration decreased ≈2-fold.	Seropositive samples defined by our in-house ELISA correlated with results from the LIAISON assay and neutralizing antibody assay.
Interiano et al, 2021 (11).	IgG responses lasted more than 120 days in children 12-19 years aged. Instead in children 0-11 years aged IgG levels significantly reduced since day 121.	Not studied.
Ireland et al, 2021 (12)	Nucleocapsid and S-RBD antibody could be detected in 78% of students for more than 6 months after infection, with a decline after 24 weeks.	Live virus neutralizing antibody titres were measured in 89 students with N-antibody. 79.8% of the students (71/89) had neutralizing antibodies. S-RBD antibodies correlated more strongly with neutralizing antibodies than nucleocapsid antibodies.
Mayanskiy et al, 2021 (13)	The seropositive rate was about 80% for at least three consecutive weeks, declining to 54% by week 18 post-exposure.	Anti-spike (anti S-RBD) antibodies, which titers correlated with the neutralization, were found in the majority of patients.
Breuer et al, 2021 (14)	In 47/54 (87%) of children there was a peak of anti-SARS-CoV-2 IgG 22–119 days after the infection; antibody levels were significantly lower after more than 4 months from the infection. Children under the age of 6 years had higher antibody levels than older children in the first 60 days after infection, followed by a more rapid decline in antibody levels.	Not studied.
Oygar et al, 2021 (15)	In 95.8% of the patients IgG were detectable up to 9 months after infection. IgG levels decreased significantly in asymptomatic and mild/moderate cases, moderately in severe/critical cases.	Not studied.
Garrido et al, 2022 (16)	IgA and IgM antibodies against the majority of antigens were detectable in the great part of children 4 months after acute infection. Levels of IgG antibodies generally increased from acute infection to 2 months after infection, showing a following decreasing 2-4 months after acute infection.	Neutralizing antibodies at a 50% inhibitory dilution (ID50) were detected in 56 of 69 (81%) cases during acute infection, 53 of 56 (95%) cases after 2 months, and 47 of 50 (94%) of cases after 4 months.
Messiah et al., 2021 (17)	96% of children with positive nucleocapside antibodies at baseline assessment continued to have antibodies more than 6 months later.	Not specifically analyzed.
Han et al, 2022 (18)	In both symptomatic and mildly symptomatic children, the positive rates of anti-S IgG, anti-SARSCoV-2 IgG, and NAb were low within 7 days after onset, but they were positive in 100% of children 14 to <28 days after onset. Antibody levels were detected up to 3 months after infection. TTe antibody GMTs during the period 14 to <56 days after symptom onset seemed to be highest in children aged 0-4 years but this data was not statistically significant.	Neutralizing antibodies showed similar kinetiks of IgG.
Tsang et al, 2022 (19)	SARS-COV-2 S-RBD IgG decreased rapidly: the average SARS-COV-2 S-RBD IgG half-life decay was 121.6 days and antibodies were detectable for 237.7 days or 7.9 months.	Not specifically analyzed.
Dowell et al, 2022 (20)	All children had persistence of humoral immunity 6 months after the infection. Antibody levels to S-RBD analyzed in 12 months-follow up were retained at a similar, although slightly reduced, level to those seen at 6 months while nucleocapsid-specific antibody levels were reduced. In 2 of 16 children (12.5%) of the 12 months-follow up there were spike-specific antibody levels below the threshold while in 4 children (25%) there was a similar loss of nucleocapsid-specific antibodies, in 1 of whom there was also a lost of the spike-specific response. The Authors showed similar activity against different viral variants.	If compared to adults, children showed higher antibody binding to SARS-CoV-2 VOC (Alpha (B.1.1.7), Beta (B.1.351) and Gamma (P.1), original Wuhan genotype) after natural infection but evidenced similar neutralizing ability.
Kinoshita et al, 2021 (21)	There was no significant difference in antibody responses to nucleocapsid and spike proteins between immunocompromised patients and controls at days 65–84 from onset of symptoms.	All 5 subjects had detectable neutralizing antibodies targeting both spike and nucleocapsid proteins of SARS-CoV-2.
Cotugno et al, 2021 (22)	Children presented high anti-Spike protein IgG levels and high concentrations of Nabs, which correlated with lower viral burdens and faster virus clearance.	There was an inverse correlation between SARS-CoV-2-specific IgG and Ab neutralization activities with viral load, virus clearance and infectivity.
Kaaijk et al, 2022 (23)	IgG serum antibody concentrations significantly decreased at 10 months compared to earlier time points in children. Despite this decline, 90% (18/20) of infected children and 89% (16/18) of infected adults remained S1-SARS-CoV-2-IgG seropositive 10 months after infection.	Not specifically analyzed.
Cohen et al, 2021 (24)	Not studied.	Not studied.

VOC, variants of concern; NAb, neutralizing antibodies; S-RBD, spike receptor-binding domain.

**Table 3:** Findings on T cellular immunity after infection.

Citation	T compartment
Tsang et al, 2022 (19)	CD4+ and CD8+ T cell responses persist over time and it is detectable even in the patient with the longest follow-up time at 219 days (who had undetectable anti-RBD IgG but persistent SARS-COV-2 specific CD4+ and CD8+ T-cell response).
Dowell et al, 2022 (20)	Cellular immune responses were detectable in 84% of children at least 6 months after infection. Compared to the cohort analysis at 6 months, T cell responses to spike were retained but somewhat reduced. Matched samples at 6 and 12 months were available for 5 of these children and were stable.
Kinoshita et al, 2021 (21)	There was no statistically significant difference between the affected patients and control groups regarding CD4+ T cell responses to actin or any of the SARS-CoV-2 proteins (membrane, envelope, nucleocapsid, or spike). Affected and control patients did not show appreciable CD8+ T cell responses. There was no significant difference in CD4+ T cell memory response for spike between affected and control patients with respect to naïve, central memory, effector memory, and terminal effector T cells.
Cotugno et al, 2021 (22)	There was a positive association between SARS-CoV-2-specific CD4 T cells and SARS-CoV2-specific IgG and Ab-neutralization activity.
Kaaijk et al, 2022 (23)	In 44% (8/18) of the infected children and 81% (17/21) of the infected adults, SARS-CoV-2-specific IFN-g + responses were detectable 10 months after symptom onset. In children at 10 months there was a 4-fold decline of S-SARS-CoV-2-specific IFN-g+ T cells at 10 months compared to frequencies at 3 weeks after infection; at 10 months there was no significant difference between exposed and unexposed children.
Cohen et al, 2021 (24)	IFNγ CD4+ and CD8+ T cell responses were significantly lower in SARS-CoV-2 infected children than in adults against the viral structural proteins, and in CD8+ T cells against ORF1ab proteins. Children’s T cells responses to polyclonal non-specific activation were also lower. T cell response in children is less antigen experienced and matured than adults. Memory T cell responses were smaller in children than in adults and this could be responsible of a weaker long-term memory response in children, with potential impact on reinfection.

**Table 4:** Humoral and cellular memory and re-infections after vaccination.

Citation	Humoral immunity: main findings	Cellular immunity: main findings	Reinfections
Frenck et al., 2021 (25)	The serum-neutralizing geometric mean titer 1 month after the 2^nd^ dose of BNT162b2 was 1283.0 in the group aged 12-15 years old and 730.8 in the group aged 16-25 years old. There was a substantial increases in the 50% neutralizing titer from baseline to 1 month after the 2^nd^ dose, equal to 118.3 in the group aged 12-15 years old and 71.2 in the group aged 16-25 years old	Not studied	Between dose 1 and dose 2 of BNT162b2, the vaccine efficacy observed was 75%. No cases of severe Covid-19 were observed. In the group 12-15 years old, after dose 2 of BNT162b2 vaccine efficacy observed was100%.
Qin CX, et al. (26)	Titers were positive in 56.8% of patients with post-V1 titers and 73.3% with post-V2 titers. Median antibody titers were 98.7 (12.9–158) U/ml and 1876 (178–2500) U/ml, respectively. Among patients with both serologies available, 16.7% had negative titers after both, 33.3% had a negative titer that became positive, and 46.7% had positive titers after both.	Not studied	During the study period two patients tested positive for SARS-CoV-2. The first patient had mild symptoms for 7 days; his post-V1serology was not available. The second patient developed infection 46 days after 2^nd^ vaccine dose with negative antibody titers.
Price A et a, 2022 (27)	Not studied	Not studied	During the delta period, among adolescents aged 12-18 years old, vaccine effectiveness against hospitalization for Covid-19 was 93% after 2-22 weeks from full vaccination and 92% in the 23 to 44 weeks after full vaccination. During the omicron period, vaccine effectiveness against hospitalization for Covid-19 was 43% in weeks 2-22 and 38% in weeks 23-44 after full vaccination. During the delta period, among adolescents aged 12-18 years old, vaccine effectiveness against critical Covid-19 was 96% as compared with 91% against hospitalization without life support. During the omicron period, vaccine effectiveness against critical Covid-19 was 79% as compared with 20% against noncritical Covid-19 During the omicron period, among children of 5-11 years old, vaccine effectiveness was 68% against Covid-19–associated hospitalization. The interval from vaccination to Covid-19 hospitalization during the omicron period was longer among 12-18 years of age group than among those 5 to 11 years of age (median, 162 days vs. 34 days).
Powell A et al, 2022 (28)	Not studied	Not studied	During delta period, after one dose of vaccine in 12-15 years old group, there was a peak (74.5%) of disease at days 14-20 after vaccination and a following decline (45.9%) at days 70-83. After 2 doses, effectiveness increased at 93.2 for delta variant. Considering 16-17 years old, vaccine effectiveness after dose one had a peak (75.9%) at days 14-10 and a gradual decline to 29.3% at days 84-14. During the omicron period vaccine effectiveness among 12-15 years old was lower: peak of 49.6% at days 14-20 after one dose, decline to 16.1% at days 70-83. After 2 doses, effectiveness increased at 83.1 for the omicron variant. For omicron the vaccine effectiveness among 16-17 years old was lower at 52,7% between days 21-27 and declined to 12.5% after day 105. Considering 16-17 years old, after two doses effectiveness increased at 96.1 for delta variant and 76.1 for the omicron variant.
Fowlkes A et al, 2022 (29)	Not studied.	Not studied.	There were more symptomatic patients among those infected by Delta variant (66%) than those by Omicron (49%). After 2 doses of vaccine, VE against any Omicron infection in children aged 5-11 years, 14-82 days after dose 2 of the Pfizer vaccine was 31%. Among adolescent aged 12-15 years, adjusted 14-149 days after dose 2 has 87% against symptomatic and asymptomatic Delta infections and 59% against omicron infection.
Burns et al, 2022 (30)	After the 2^nd^ dose of vaccine there was an important generation of anti-wild type and anti-Omicron antibodies if compared to the pre-vaccination period. antibodies against Omicron RBD increased after the 2^nd^ dose, but not after the 1^st^ dose, of vaccine. There was a significant loss of all anti-SARS-CoV-2 antibody responses by six months, with antibody responses decreased to levels comparable to titers seen at the V1 time point, following the first vaccine dose.	Not studied.	Not studied.
Chen et al, 2022 (31)	All recovered COVID-19 patients and vaccine recipients had an MN titer of ≥10 against the ancestral lineage A virus. In a similarl way, 94.1% of vaccine recipients and all recovered patients had MN titer of ≥10 against the Beta variant. There was a n MN titer of ≥10 against the Omicron variant in only 38.2% and 26.7% of vaccine recipients and recovered patients respectively.	Not studied.	Not studied.
Haskin et al, 2021 (32)	There ws a positive serologic response (63%) at a median of 37 days after the 2^nd^ vaccine dose. After vaccination, there was an antibody levels peak at 1–2 months after the 2^nd^ vaccine dose. A higher proportion of patients developed a positive serologic response after infection than after COVID- 19 vaccination (100% vs 63%).	Not studied.	None of the study participants developed symptomatic Covid-19, but asymptomatic infections were not assessed.

### Duration of Immune responses after infection

Fifteen studies (9–23) analyzed duration of humoral immunity after SARS-CoV-2 natural infection (**Table 2** for further details). All these studies documented a seroconversion in the majority of children who have been infected with SARS-CoV-2. The duration of follow up after infection was different, ranging from a minimum of three months (18) to a maximum of twelve months (20). Two studies (11, 14) found different duration of immunity in children of different ages. In particular, Interiano et al. (11) reported longer persistence of IgG responses in children aged 12-19 rather than in those aged 0-11 years. Conversely, Breuer et al. (14) evidenced higher and more lasting antibody levels in children younger than 6 years. One study (15) underlined different duration of immunity related to a different severity of infection: IgG levels decrease more significantly in patients with mild/moderate infection rather than in severe/critical ones. Only one study (21) compared the duration of immunity in immunocompetent and immunocompromised patients, showing no significant differences. Seven studies (10, 12, 13, 16, 18, 20–22) made comments on neutralizing antibodies. Only one study (20) evaluated the humoral immunity developed against viral variants, showing similar activity against different variants.

Six (19–24) studies analyzed cellular immunity developed after SARS-CoV-2 infection. Only one study comments about B cell compartment and it found that SARS-CoV-2 specific B cells are positively associated with anti-SARS-CoV-2 IgG and neutralization activity (22). On the other hand, the same studies (19–24) reported the development of a specific T cell response after infection in children. Three studies (19, 20, 23) also analyzed duration of specific T cell immunity, reporting detectable T cell response for six to twelve months after initial infection. Two studies (23, 24) reported differences in duration of T cell immunity between adults and children; in particular, T cell responses seemed to be significantly lower and faster in decline in children than in adults infected by SARS-CoV-2. Only one study (20) reported data about activity against viral variants, showing a significant cross-reactivity of T cell compartment between variants. Further details are available in **Tables 1**–**3**.

### Duration of immune responses or cases of reinfections after vaccination

Eight studies discussed humoral and cellular memory and re-infections after SARS-CoV-2 vaccination. In six studies (25, 27, 29–32), paediatric population received BNT162b2 (Comirnaty, Pfizer-BioNTech), and in the remaining two studies (26, 28) received BNT162b2 (Comirnaty, Pfizer-BioNTech) or mRNA-1273 (Spikevax, Moderna). There are five clinical studies (25, 26, 30–32) which analyzed the duration of immune responses after SARS-CoV-2 vaccination. None of them examined the duration of cellular immunity in the paediatric population. One study highlighted that 12-to-15-year-old recipients have greater neutralizing antibodies titers after second dose of vaccination relative to 16-to-25-year-old participants (25). Robust humoral immunity after only two doses of vaccine was shown (26, 30). Prior immunity may matter, as shown in a separate study, after a third vaccination, 100% of those with prior positive responses increased or have preserved maximum antibodies titers, compared to 54.5% with prior negative response (26). This immunity may not last long, as loss of all anti-SARS-CoV-2 antibody responses was shown in one study 6 months after vaccination (30). Two studies compared serologic response after infection and after SARS-CoV-2 vaccination, showing that recovered COVID-19 patients had a higher serum neutralization antibody titer compared to vaccinated participants (31, 32). Moreover, antibody levels among COVID-19 positive patients continued to remain high, even 6–8 months following the infection (32).

Six studies analyzed efficacy of SARS-CoV-2 vaccines in preventing reinfections and infection caused by other variants, like Delta and Omicron variant (25–29, 32). Different studies showed that there were no cases of infection by SARS-CoV-2 with an onset at 7 or more days after the second dose of BNT162b2 recipients (25, 32). In this paediatric population only a few cases of mildly symptomatic infection that did not require hospitalisation have occurred (26). The efficacy of the vaccine differs according to variants of the virus, being higher for Delta variant rather than for Omicron variant in 12-15-years-old and 16-17-years-old children (28). Two doses of Pfizer- BioNTech vaccine were effective in preventing 87% infection by Delta variant and 59% by Omicron variant (29). Morover, BNT162b2 vaccination reduced the risk of Omicron-associated hospitalisation by two thirds among children 5-to-11-year-old and registered a lower protection against Omicron-associated rather than Delta-associated hospitalisation among adolescents 12-to-18-year-age (27). Further details are available in **Tables 1** and **4**.

### New key publications released after the systematic review

Importantly, a number of relevant studies have been published after the end of study search of our systematic review to further understand current scenarios of protection of children after infection or vaccination, which might be useful to understand how to deal with current pre-Omicron and Omicron waves.

Di Chiara et al. assessed anti–SARS-CoV-2 spike receptor-binding domain (S-RBD) IgG kinetics in 697 patients with mild/asymptomatic SARS-CoV-2 infection, including 351 children or older siblings and 346 parents, up to one year after initial infection (33). Children had significantly higher S-RBD IgG titers than older patients across all follow-up time points, and longitudinal analysis of 56 study participants sampled at least twice during follow-up demonstrated the persistence of antibodies up to 10 months from infection in all age classes, despite a progressive decline over time.

Khaitan et al,. as part of the DISCOVER study (34), measured neutralizing Abs (nAbs) and IgG Abs to SARS-CoV-2 Ags and spike protein variants in symptomatic and asymptomatic children. They found that children developed nAbs that remained detectable longer than in adults but waned in titer over time and broad IgG Abs that also waned in level over time. Moreover, nAb levels were lower postinfection than those reported after immunization, suggesting that children might benefit from a booster as adults do (35).

Tang et al. in the USA analyzed virus-neutralizing capacity against SARS-CoV-2 Alpha, Beta, Gamma, Delta and Omicron variants in 177 paediatric patients hospitalised with severe acute COVID-19, MIS-C, and mild COVID-19 during 2020 and early 2021 (36). They found that children retained neutralizing antibodies in the convalescent post-acute phase, but it tended to decrease with time, and the neutralizing activity against Omicron was poor in all age groups. Conversely, vaccination induced a broader neutralizing antibody response against VOCs in naïve children compared with the natural immunity following SARS-CoV-2 infection (36).

Bartsch et al. investigated vaccine-induced humoral immune response in 6-to-11-years-old children and seems to confirm the importance of booster dose in previously infected paediatric population. Indeed vaccinated children elicited robust cross-VOC antibody responses and a 100 μg doses resulted in higher preserved Omicron-specific functional humoral immunity rather than those with diagnosed of COVID-19 or MIS-C (37). Importantly, a South Korean population cohort study of 3.2 million adolescents identified 29285 SARS-CoV-2 infection, 11 of which were classified as critical. None of these critical cases were vaccinated and overall estimate of vaccine effectiveness of two doses against preventing infection was 75.5% to 80.4% in the immediate post-vaccination month, but this waned to near 40% over two months post-vaccination (38). Lin and colleagues provided further data on the protection against hospitalization related to omicron variant in children 5 to 11 years of age who had receives vaccination and had had previous SARS-CoV-2 infection (39).

## Discussion

SARS-CoV-2 infection frequently leads to mild or asymptomatic infections in children, although in rare cases they can develop critical acute disease (40), a severe post hyperinflammatory syndrome (MIS-C) (41) or even Long Covid (42). Although the causes are not yet fully established, current evidence suggested that more efficient innate (43) and local tissue responses, better thymic function, cross-reactive immunity (44), different expression of Treg cells (45), and better nasal immunity (46) can play a role in this observed age-dependent risk of severe disease. Although these mechanisms may explain differences in disease expression, duration of immunity against new SARS-CoV-2 infections in children previously infected or vaccinated remains poorly understood. In fact, while social events, interactions, relationships and being in close environments (such as schools) may theoretically provide a perfect setting for COVID-19 transmission, all these aspects are of critical importance for appropriate child development. Not by chance, prolonged lockdowns and school closures have had a catastrophic negative impact on child wellbeing worldwide (47). Therefore, a critical appraisal of current evidence in term of duration of SARS-CoV-2 immunity in children is mandatory and might offer physicians, policy makers and parents appropriate information to implement the most balanced decisions that, at the same time, protect children from consequences of COVID-19 but also allow them to live all the necessary experiences for their proper growth. In this regard, our systematic review on duration of immunity in children previously infected or vaccinated, the first one to our knowledge, provides important and practical information. Specifically, studies addressing duration of humoral and cellular immunity suggested that, *in vitro*, children retained appropriate memory and cross-reactive protection against pre-Omicron strain for about 10-12 months, although only a few studies focused on cellular memory and none included also information about clinical reinfections. Studies evaluating protection after vaccination suggested that children developed short protection against asymptomatic or mild infection but maintained good protection against severe/critical disease. Unfortunately, the number of available studies is limited and no data about long-term protection after both natural infection and vaccination in children are available.

It is important to highlight that the available studies addressing duration of immunity after SARS-CoV-2 infection mostly focused on immunological (either humoral or cellular) parameters, and only a few addressed the real-world impact of reinfections in children. The first study was published by Mensah et al. in the UK, where they prospectively evaluated risk of SARS-CoV-2 reinfection in children and compared this with the risk in adults during Delta and pre-Delta periods (48). Overall, they found that children had a lower risk of reinfections compared with adults (72.53 per 100 000 vs 21.53 per 100 000), and a similar rate of hospitalisations or pediatric intensive care unit (PICU) admissions in children during the first or second infection (2.7% and 2.4% hospitalisations during first and second episode, and seven and four PICU admissions, respectively). A similar preprint from Israel during Delta period showed that previously infected children remained protected against reinfection to a high degree for 18 months, although in this case severity of reinfections was not reported (49).

More recent studies, although supportive of comparatively longer period of immunity after natural infection, continue to support vaccinating children with prior immunity from pre-Omicron strains to maximize protection against Omicron and potential future strains (36–38, 50–52).

Less information is available about protection in children already infected with Omicron, although three recent preprints from Portugal (53), Qatar (54) and Denmark (55) that included both adults and children suggested that infection with Omicron provided substantial protection against reinfection (56). Importantly, these studies have been performed in countries that implemented impactful vaccination campaigns and, therefore, it is probable that such positive findings were due to an immune cross-stimulation by both vaccinations and Omicron infection, rather than Omicron alone. In addition, these studies did not include immunological parameters. Conversely, Dowell et al. from the UK recently released a preprint antibody and cellular immune response following Omicron infection in children aged 6-14 years and related this to prior SARS-CoV-2 infection and vaccination status (57). Interestingly, primary Omicron infection elicited a weak antibody response in 53% of children, while a secondary Omicron infection following prior infection induced better neutralizing antibodies. Importantly, vaccination elicited the highest levels of antibody response and was also strongly immunogenic following prior natural infection with Omicron. Although cellular responses against Omicron were strong in all children infected with Omicron, this study further reinforced the hypothesis that a vaccine booster after natural infection could be an appropriate precautionary strategy to discuss with families, awaiting further evidence and new scenarios particularly for children at highest risk of severe COVID-19, MIS-C or Long COVID.

Our study has limitations to address. First, studies on the topic are heterogeneous and mostly focused on only a part of the immunological or clinical data that can give a better overview of the real protection of children after a previous SARS-CoV-2 infection or vaccination. For example, most studies focused on duration of humoral immunity while a minority also included cellular immunity, non of them having long-term follow-up to address if those patients with evidence of humoral/cellular memory were protected from symptomatic infections. Secondly, duration of follow-up was variable, ranging from weeks to 12-18 months. Third, most studies were performed during the pre-Omicron era and a minority addressed cross-protection against pre-Omicron and Omicron variants. Therefore, the information provided does not allow to derive firm conclusions, but mostly a guidance awaiting more solid evidence. Fourth, neutralizing activities were specifically studied in a minority of studies and usually on a subgroup of the originally enrolled studies. Fifth, no conclusion con be drawn about protection of children with comorbidities after infection or vaccination because studies included patients with great variability of pathological condition, although our findings were encouraging. Importantly, one recent study coordinated by an Italian group showed that children with inflammatory bowel disease with distinct immune suppressive treatment had a good safety and immunogenicity profile following SARS-CoV-2 mRNA vaccination (58). While perinatally infected HIV children or previously transplanted patients had a different immunological response which suggested their eligibility for booster doses (59, 60). However, the paucity of data and differences in study population do not allow to derive firm conclusions. Importantly, no studies so far have evaluated the role of immune memory from previous infection or vaccination in preventing long covid after re-infections, while a few studies have suggested that previous memory can be associated with a lower risk of developing MIS-C (61, 62).

In conclusion, the analyzed literature suggested that most children infected during the pre-Omicron era developed long lasting (at least 10-12 months) humoral and cellular immunity against pre-Omicron SARS-CoV-2 variants, but have reduced *in vitro* cross-reactivity against Omicron. These data seem in line with the two studies addressing re-infections after previous COVID-19, showing a low but possible risk of new infections in children infected before Omicron, with a risk of a similar or lower disease severity compared to previous infection. Conversely, although vaccination had a limited efficacy in preventing new infection with Omicron, *in vitro* studies suggested that vaccine-induced immunity provided better *in vitro* cross-neutralization against Omicron.

All together, these data suggested that, until better evidence is available and given difficulties in predicting future variations of SARS-CoV-2 and their impacts (63, 64), it might be reasonable to offer families of children infected before Omicron a booster vaccination, probably not earlier than six months since previous infection. For those with a known infection during Omicron, a more cautious approach could be suggested. Since adult and preliminary paediatric studies suggested that vaccination may induce a general better cross-variant neutralization also in those infected with Omicron, for more fragile children a booster vaccination should be considered, pending proper discussion with the family. However., it is important to highlight that the effect of booster in providing better neutralization protection against new post-Omicron variants is still unknown.

Importantly, in such a rapidly evolving scenario, new studies addressing the protection against new variants, even in children infected with Omicron, are urgently needed.

## Data availability statement

The original contributions presented in the study are included in the article/**Supplementary Material**. Further inquiries can be directed to the corresponding author.

## Author contributions

DB and SE conceptualized the study. LC, CG, FC and FB performed data collection and data synthesis. DB, LC, CG, FC and FB wrote the first draft of the manuscript. DB and SE revised and wrote the final draft of the manuscript. SE supervised the manuscript. All authors contributed to the article and approved the submitted version.
