# Supplementary material for: Duration of immunity to SARS-CoV-2 in children after natural infection or vaccination in the omicron and pre-omicron era: A systematic review of clinical and immunological studies
Source: Front Immunol. 2023 Jan 11;13:1024924. doi: 10.3389/fimmu.2022.1024924 (PMC9874918; doi:10.3389/fimmu.2022.1024924)
Supplement: Supplementary file 1 [file DataSheet_1.pdf]

Supplementary material:

Supplementary table S1. PRISMA checklist

Section/topic	#	Checklist item	Reported on page #
**TITLE**			
Title	1	Identify the report as a systematic review, meta-analysis, or both.	#1
**ABSTRACT**			
Structured summary	2	Provide a structured summary including, as applicable: background; objectives; data sources; study eligibility criteria, participants, and interventions; study appraisal and synthesis methods; results; limitations; conclusions and implications of key findings; systematic review registration number.	#2
**INTRODUCTION**			
Rationale	3	Describe the rationale for the review in the context of what is already known.	#3
Objectives	4	Provide an explicit statement of questions being addressed with reference to participants, interventions, comparisons, outcomes, and study design (PICOS).	#3
**METHODS**			
Protocol and registration	5	Indicate if a review protocol exists, if and where it can be accessed (e.g., Web address), and, if available, provide registration information including registration number.	#4
Eligibility criteria	6	Specify study characteristics (e.g., PICOS, length of follow-up) and report characteristics (e.g., years considered, language, publication status) used as criteria for eligibility, giving rationale.	#4
Information sources	7	Describe all information sources (e.g., databases with dates of coverage, contact with study authors to identify additional studies) in the search and date last searched.	#4
Search	8	Present full electronic search strategy for at least one database, including any limits used, such that it could be repeated.	#4 and suppleme ntary
Study selection	9	State the process for selecting studies (i.e., screening, eligibility, included in systematic review, and, if applicable, included in the meta-analysis).	#5
Data collection process	10	Describe method of data extraction from reports (e.g., piloted forms, independently, in duplicate) and any processes for obtaining and confirming data from investigators.	#5
Data items	11	List and define all variables for which data were sought (e.g., PICOS, funding sources) and any assumptions and simplifications made.	#5
Risk of bias in individual studies	12	Describe methods used for assessing risk of bias of individual studies (including specification of whether this was done at the study or outcome level), and how this information is to be used in any data synthesis.	#5
Summary measures	13	State the principal summary measures (e.g., risk ratio, difference in means).	#6
Synthesis of results	14	Describe the methods of handling data and combining results of studies, if done, including measures of consistency (e.g., I^2) for each meta-analysis.	#6
Risk of bias across studies	15	Specify any assessment of risk of bias that may affect the cumulative evidence (e.g., publication bias, selective reporting within studies).	#6
Additional analyses	16	Describe methods of additional analyses (e.g., sensitivity or subgroup analyses, meta-regression), if done, indicating which were pre-specified.	Not performed

RESULTS			
Study selection	17	Give numbers of studies screened, assessed for eligibility, and included in the review, with reasons for exclusions at each stage, ideally with a flow diagram.	#6
Study characteristics	18	For each study, present characteristics for which data were extracted (e.g., study size, PICOS, follow-up period) and provide the citations.	Table 1
Risk of bias within studies	19	Present data on risk of bias of each study and, if available, any outcome level assessment (see item 12).	Suppleme ntary material
Results of individual studies	20	For all outcomes considered (benefits or harms), present, for each study: (a) simple summary data for each intervention group (b) effect estimates and confidence intervals, ideally with a forest plot.	Table 1 and Suppleme ntary materiial
Synthesis of results	21	Present results of each meta-analysis done, including confidence intervals and measures of consistency.	#6-9 and tabs 1-4
Risk of bias across studies	22	Present results of any assessment of risk of bias across studies (see Item 15).	Suppleme ntary material
Additional analysis	23	Give results of additional analyses, if done (e.g., sensitivity or subgroup analyses, meta-regression [see Item 16]).	Not performed
**DISCUSSION**			
Summary of evidence	24	Summarize the main findings including the strength of evidence for each main outcome; consider their relevance to key groups (e.g., healthcare providers, users, and policy makers).	#9-12
Limitations	25	Discuss limitations at study and outcome level (e.g., risk of bias), and at review-level (e.g., incomplete retrieval of identified research, reporting bias).	#12-13
Conclusions	26	Provide a general interpretation of the results in the context of other evidence, and implications for future research.	#13
**FUNDING**			
Funding	27	Describe sources of funding for the systematic review and other support (e.g., supply of data); role of funders for the systematic review.	No

Adapted From www.prisma-statement.org. and"Moher D, Liberati A, Tetzlaff J, Altman DG, The PRISMA Group (2009). Preferred Reporting Items for Systematic Reviews and Meta-Analyses: The PRISMA Statement. PLoS Med 6(7): e1000097. doi:10.1371/journal.pmed1000097"

Supplementary Table S2. Search strategy and results of PubMed database

Search number	Search Details	PubMed Results
**1**	duration sars-cov-2 immunity children	112
**2**	children immune responses sars-cov-2	1508
**3**	children immune responses sars-cov-2 vaccine	486
**4**	duration immune responses sars-cov-2 and children	58
**5**	cellular immunity after SARS-CoV-2 children	50
**6**	humoral immunity after SARS-CoV-2 children	54
**7**	humoral immunity after SARS-CoV-2 vaccine children	28
**8**	Cellular immunity after SARS-CoV-2 vaccine children	21
**9**	SARS-CoV-2 reinfections children	103

Supplementary Table S3. Study limitations

**Citation**	Country	Limitations
Bloise et al, 2021	Italy	small sample size; children with mild infection, not requiring hospitalization; lack of discrimination between antibodies against N and S protein of SARS-CoV-2.
Toh et al, 2021	Australia	small sample size; only a subset of samples was available for the microneutralization assay; the role and durations of other components of the immune system, (such as the cellular and mucosal responses) during SARS-COV-2 infection remain undetermined.
Interiano et al, 2021.	USA	Small number of patients and only serological studies
Ireland et al, 2021	UK	inability to confirm acute SARS-CoV-2 infection in symptomatic participants prior to recruitment); most seropositive children in June 2020 were reported by their parents to be asymptomatic and, therefore, the timing of their infection was not known; exclusive assessment of the correlation of N and RBD antibodies with neutralising activity during the first two rounds of testing in June and July 2020; no analyses of cellular immune responses; not possible comment on the protective effects of prior SARS-CoV-2 infection against reinfection with new variants).
Mayanskiy et al, 2021	Russia	Small sample size, only serological studies
Breuer et al, 2021	Israel	The single-site sample collection may have led to a geo-demographic population bias, as seroprevalence in other regions with different rates of infection may have been different. we did not find that opening schools increased the infection rates among children.
Oygar et al, 2021	Turkey	not obtain serial blood samples from all children enrolled in the study since most of them were young and frightened. Furthermore not determine the responses to IgG before 14 days, and we could not test IgM levels since we chose to test IgA levels.
Garrido et al, 2022	USA	The study not include participants with severe COVID-19 or children with MIS-C. Also they did not evaluate cellular immunity, which is likely required for long-term immunological memory, or systemic inflammatory responses. They only have data for children and adolescents up to 4 months after infection.
Messiah et al 2021	USA	Authors unable to confirm COVID-19 infection before the baseline assessment, thus these data cannot confirm durability beyond 7 months. 57.9% of the sample were negative for infection-induced antibodies at their third measurement point, suggesting a significant proportion of children are still immune-naïve to SARSCoV-2 because of natural infection. Only serological studies performed
Han et al, 2022	South Corea	Few children had their blood drawn >56 days afer onset, and paired samples were not obtained from all children, limiting long-term analysis and interpretation of the antibody kinetics. The antibody titers obtained in this study more refect the titers at diferent time points in a heterogenous pediatric population with COVID-19. Moreover, we did not compare the antibody responses between asymptomatic and symptomatic children because determining the start of SARS-CoV-2 infection in asymptomatic children was not possible. Severe cases of COVID-19 were also not included in this study
Tsang et al, 2022	China	small sample; duration of follow-up limited and unevenly distributed; only SARS-COV-2 anti-RBD, which targeted the S1 domain of the spike protein, was investingated; other subsets of T cell responses (such as Tr, Tfh) to SARS-COV-2 peptide pools were not evaluated
Dowell et al, 2022	UK	Only alfa and beta period, comparisons with adults limited in some time points
Kinoshita et al, 2021	USA	Small sample size
Cotugno et al, 2021	Italy	small sample sizes within each comparison; patients are from one region reduces translation; (3) the lack of adjustment for multiple comparisons, as this was a descriptive study of a unique cohort; and (4) the inability to accurately estimate timing of infection in asymptomatic patients; (5) short follow-up

Kaaijk et al, 2022	Netherlands	Small number of children; T immunity not assessed after in vitro stimulation with variants
Cohen et al, 2021	China	Small number of children; T immunity not assessed after in vitro stimulation with variants; short f-up
Frenck et al 2021	USA	No data on long-term efficacy (7 days after 2nd dose) and safety (one month after 2nd dose); 2) The efficacy analysis was prespecified as descriptive because an accurate sample size to assess vaccine efficacy could not be calculated before the start of the trial, given uncertainties about the incidence of SARS-CoV-2 infection.
Qin CX, et al	USA	small sample
Price A et a, 2022	USA	They estimated effectiveness only for the BNT162b2 vaccine, which was widely available for adolescents 12 to 18 years of age in the United States.<br>Because of the recent authorization of the BNT162b2 vaccine for children 5 to 11 years of age in the United States, the sample and the duration of follow-up since full vaccination were limited. Misclassification due to reduced sensitivity of the SARS-CoV-2 assay cannot be ruled out; They could not evaluate vaccine effectiveness after a booster dose
Annabel et al, 2022	UK	Only assessed microbiologically confirmed reinfections, not immunological studies
Ashley et al, 2022	USA	do not include analyses of VE against asymptomatic infection and symptomatic infection at this time; no immunological studies
Burns et al, 2022	USA	Analyses limited to anti Spike antibodies
Chen et al, 2022	China	did not assess cross-reactive T cell immunity against the Omicron variant; since COVID-19 vaccine was not yet recommended for children aged 11 or younger in Hong Kong at the time of writing this manuscript, we were not able to assess the effect of Omicron variant in this age group. this study only included BNT162b2 vaccine recipients.
Haskin et al, 2021	Israel	No evaluation of cellular immunity in response to vaccination and small sample size. Also lack of an appropriate control group of healthy vaccinated individuals. Not test for anti–COVID-19 antibodies before vaccine administration to identify patients with previous yet unknown COVID-19 infection.
